# Evaluating and excluding swap errors in analogue tests of working memory

**DOI:** 10.1038/srep19203

**Published:** 2016-01-13

**Authors:** Paul M Bays

**Affiliations:** 1 University of Cambridge, Department of Psychology, Downing St, Cambridge, UK

## Abstract

When observers retrieve simple visual features from working memory, two kinds of error are typically confounded in their recall. First, responses reflect noise or variability within the feature dimension they were asked to report. Second, responses are corrupted by “swap errors”, in which a different item from the memory set is reported in place of the one that was probed. Independent evaluation of these error sources is vital for understanding the structure of internal representations and their binding. However, previous methods for disentangling these errors have been critically dependent on assumptions about the noise distribution, which is *a priori* unknown. Here I address this question with novel non-parametric (NP) methods, which estimate swap frequency and feature variability with fewer prior assumptions, and without a fitting procedure. The results suggest that swap errors are considerably more prevalent than previously appreciated (accounting for more than a third of responses at set size 8). These methods also identify which items are swapped in for targets: when the target item is cued by location, the items in closest spatial proximity are most likely to be incorrectly reported, thus implicating noise in the probe feature dimension as a source of swap errors.

A number of recent advances in understanding the structure of working memory^1^,^2^ have been driven by results from continuous recall tasks, in which memory stimuli are chosen uniformly at random from an analogue feature dimension, e.g. orientation, position, or colour hue^3^,^4^,^5^,^6^,^7^,^8^. In the most common version of this task (Fig. 1), observers are asked to reproduce from memory one element of a visual array, indicated by a probe in a second feature dimension (often, though not exclusively, location). In comparison to the older *change detection* methodology^9^,^10^, which provides only a binary (correct/incorrect) measure of performance, this *delayed estimation* task^4^ gives access to both the direction and magnitude of an observer’s error with each response.

One of the goals of studies using this paradigm has been to identify the distribution of errors in memory along the reported feature dimension, observe how this varies with experimentally-controlled factors, and thereby draw conclusions about the internal representation and maintenance of the reported feature (e.g. the fact that the width of the error distribution for many simple visual features increases continuously with set size has been used to argue for *resource-based* models of working memory^5^,^8^,^11^,^12^,^13^). However, interpreting the pattern of responses on this task is made challenging in multi-item arrays by the possibility of *swap errors*^8^, in which an observer reports an item in the array other than the one that was probed. Qualitatively, the presence of such errors is often discernable in data as a central tendency in the deviation of responses from *non-target* feature values, which would otherwise be uniform. (Note, the term *swap error* refers to the fact that a non-target feature is “swapped in” for the target feature. It should not be taken to imply a symmetrical exchange of places. These errors are also referred to as *non-target*, *binding*, *misbinding*, *transposition*, or *intrusion errors*.).

Because their distribution with respect to the target feature is random, swap errors distort estimates of memory precision, and may be mistaken for random guesses if only the raw deviation of responses from the target is examined. Reliable methods of excluding the contribution of swap errors from empirical response distributions are therefore of critical importance.

Furthermore, swap errors are of interest in their own right, as representative of failures in *feature binding*, i.e. grouping features together into visual objects^14^,^15^,^16^. Failures of binding in working memory have been identified as markers of cognitive aging^17^,^18^, dementia^19^, and Parkinson’s disease^20^, and there is evidence that binding is selectively impaired by medial temporal lobe lesions^21^. While the principles by which elementary visual features are represented in neural populations are well understood, the nature of binding representation is still largely unknown. Progress on this question will almost certainly require accurately evaluating misbinding in behavioural responses.

Existing methods for evaluating swap error frequency^8^,^11^ are based on maximum-likelihood (ML) principles, and require specifying a model of how errors are distributed within memory for the reported feature dimension, i.e. precisely the information we hope to extract from the data. The original and most widespread formulation^8^, followed previous work^6^ by modelling errors in the report dimension with a normal distribution centred on the target feature value, plus a uniform component to capture random responses. A number of other models have subsequently been put forward^11^,^12^,^13^, each claiming to provide a superior account of this distribution, and their relative merits are strongly debated. Meanwhile, estimates of swap frequency obtained using this method have varied considerably, with many studies finding them prevalent, some claiming not to find them at all. A recent meta-analysis^22^ concluded that swap errors occur but account for only a small fraction of responses (2.35% per non-target item, for a total of 16.5% at set size 8).

Here, I show that the accuracy of ML estimates of swap frequency depends critically on correctness of the model assumptions about errors in the report dimension. I propose instead a new class of non-parametric (NP) methods that estimate swap error frequency without any assumptions about the distribution of errors in the reported dimension. Re-examining data from nine published studies of delayed report, I find evidence that previous methods may have significantly underestimated the frequency of swap errors, particularly in larger arrays. I further show how the underlying distribution of errors in the reported dimension can be recovered from observers’ responses, with implications for current debates about working memory capacity. Finally, I use these new methods to probe the basis of swap errors, showing that—for location-based probes—proximity to the target item strongly influences the probability of non-target report. This identifies variability in memory along the *probe* feature dimension as a likely basis for these errors.

## Results

**Figure 1:**
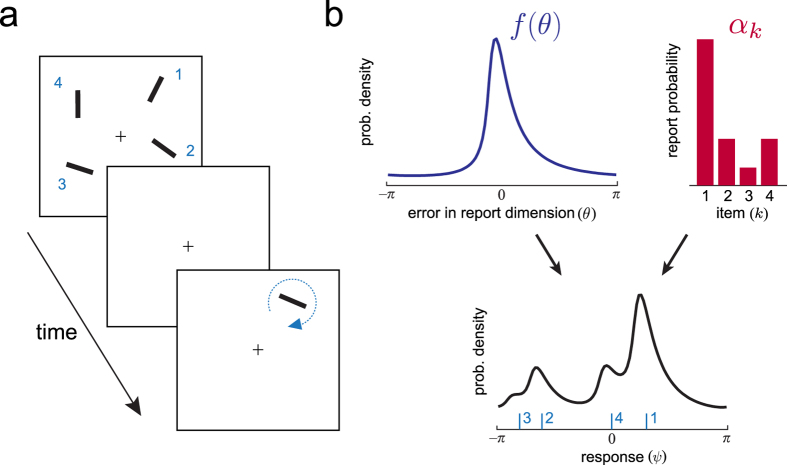
Delayed report task and generative model. (**a**) Procedure of a typical delayed report task. In this example, the feature dimension for report is orientation, and the probe dimension is location. Observers adjust the probe bar to match the remembered orientation at the same location (numbers and symbols in blue are for illustration and were not present in the display). (**b**) A model of responses on the task. Memory for report features (orientations) is distributed around true values according to a probability function 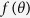. Each item (*k*) is chosen for report with probability 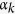. Incorrectly reporting memory of one of the items 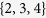 not in the probe location constitutes a *swap error*, and occurs with probability 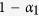. The bottom panel illustrates the resulting probability distribution of the reported orientation in the example trial shown in (**a**). Note that axis limits at 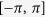 reflect the circular space of possible responses.

The task procedure presented in Fig. 1a exemplifies the delayed report design. Observers are required to reproduce a visual feature (here, orientation) belonging to one item, indicated by a probe, from a previously-presented array containing one or multiple items (here, four) with randomly-selected features. A minimal description of the process of response generation is illustrated in Fig. 1b. Each item *k* in the array has a probability of being reported, 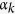. Each reported feature has some probabilistic error relative to its true value, corresponding to a distribution 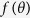. Responses follow a mixture distribution, reflecting both these factors. Note that this description is agnostic as to the presence or absence of random responses or guesses (e.g.^6^) which will manifest as a uniformly-distributed component in the report-dimension distribution 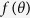.

We would like, based on responses from a sequence of such trials, to disambiguate errors due to reporting the wrong item (swap errors, which will occur if 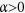 for one or more unprobed items) from errors due to variability in report of the correct item (occurring for any 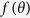 with width 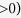. Previous studies have addressed this question using maximum-likelihood (ML) methods to fit models incorporating swap errors to experimental data. However, this necessitates specifying a particular parametric form for 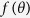, and the correct choice for this distribution—which is closely related to the underlying architecture of working memory—is strongly debated.

**Figure 2:**
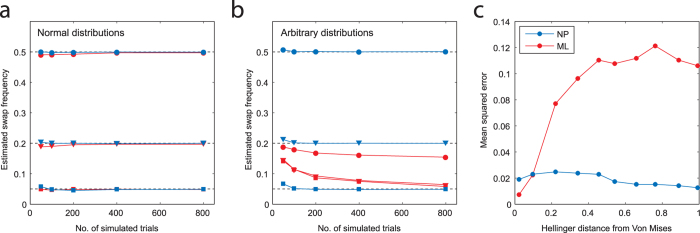
Comparison of maximum-likelihood (ML) and non-parametric (NP) estimates of swap error frequency, based on simulated recall data (see Methods). (**a**) Simulations in which errors in the report dimension 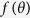 are drawn from a circular normal (Von Mises) distribution. Fitting a model based on normally-distributed errors^8^ provides accurate estimates of swap frequency, even with few trials (mean estimates in red; true frequencies: 0.5, circles; 0.2, triangles; 0.05, squares). A non-parametric method that makes no such assumption about 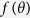 also accurately estimates swap frequency (blue). (**b**) Simulations with arbitrary (randomly-generated) distributions 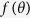. The model based on normally-distributed errors now provides very poor estimates of swap frequency, while the non-parametric method rapidly converges to the true frequency. (**c**) Estimation error as a function of similarity of 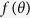 to Von Mises. The ML method provides accurate estimates of swap frequency only when the model of errors in the report dimension is matched. Accuracy of the NP method is largely independent of this distribution.

The original and most widespread ML method to incorporate non-targets^8^ specifies a circular normal (Von Mises) error distribution, centred on the true feature value, plus a uniform component intended to capture random responses. To illustrate the importance of choosing a correct parameterization for 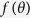, Fig. 2 plots (in red) results of fitting this ML model to simulated data in which 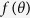 matched (Fig. 2a) or did not match (Fig. 2b,c) these assumptions of the model (see Methods for full details). These results show that the reliability of ML estimates of swap error frequency depend critically on correctness of the model of errors in the report dimension: mean squared error (MSE) increased rapidly as errors deviate from the expected distribution (Fig. 2c; MSE for normal 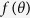, 0.003; for most strongly non-normal 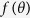, 0.11; unreliable estimates were obtained for distributions with both positive and negative kurtosis; see Supplementary Figure S1 for estimates of the target component). When the generative model was mismatched with ML assumptions, the error in the ML estimate of swap error frequency was correlated with the magnitude of the estimated uniform component (r = −0.49, p < 0.001), suggesting that the ML method systematically mistook swap errors for random errors.

In contrast to previous approaches, the non-parametric (NP) method proposed here makes no *a priori* assumptions about the form of 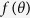. As a result, its estimates of swap frequency (plotted in blue in Fig. 2a–c) are robust to changes in the distribution of errors in the report dimension (MSE for normal 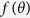, 0.015; for most strongly non-normal 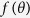, 0.013). Proofs of consistency (i.e. that NP estimates necessarily converge in probability to the true value) can be found in Methods. See Supplementary Figure S2 for analyses of bias and variance. NP estimates also have the advantage of being obtained without a fitting procedure. MATLAB code for these methods will be made available online at www.bayslab.com.

The NP method draws on the concept of the *mean resultant vector* of a distribution on the circle. The mean resultant vector has the following key properties: (1) the direction (argument) of the resultant is equal to the mean of the distribution, and its length (modulus) is inversely related to the distribution’s variance; (2) the resultant for a mixture of distributions is the weighted sum of the resultants for the component distributions, with weights equal to the mixture proportions; (3) the resultant of a uniform distribution has length zero.

**Figure 3:**
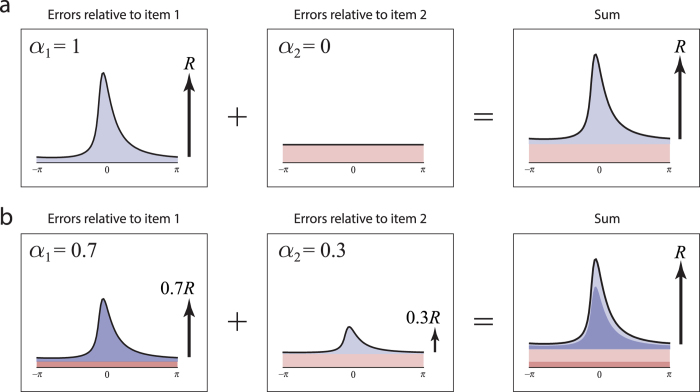
Illustration of the NP method. (**a**) Error distributions calculated relative to each of two items in an illustrative situation where all reports are of item 1 (i.e. no swap errors). The distribution of responses relative to item 1 (left) is equal to 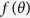 and the resultant length (arrow) is 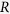, the resultant length of 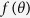. The distribution relative to item 2 (centre) is uniform, and the resultant length is zero. The sum of the two distribution functions (right) has resultant length *R*, and 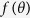 can trivially be recovered by subtracting a uniform distribution from this sum. (**b**) Error distributions where reports are distributed unevenly between items. Each error distribution is now a mixture of 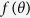 (blue) and the uniform distribution (red). The uniform components do not contribute to the resultant length, which is in each case proportional to the mixture parameter 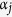. The sum of the two distribution functions (right) is identical to that in (**a**). The resultant length of the sum is the resultant length of 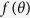, and 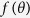 itself can again be recovered by subtracting a uniform distribution from the sum. The mixture parameters can be calculated as the ratio of the individual resultant lengths (left, centre) to the resultant length of the sum (right). These calculations form the basis of the NP estimates.

Because items are uniformly and independently distributed, the expected error distribution around probed items is a mixture of the report-dimension error distribution 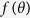, with mixture parameter equal to the proportion of trials 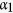 on which the probed item is reported, and a uniform distribution, with mixture parameter equal to the proportion of trials 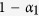 on which an unprobed item is reported. The resultant length of this mixture distribution is a fraction 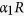 of the resultant length *R* of 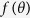. In general the resultant length of the distribution of responses relative to the *k*th item has expected value 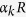, and the sum of all such resultants has expected value *R*. So the ratio of the resultant for the probed item and the sum of resultants for all items has expected value 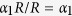. This forms the basis for an estimate of swap error frequency (see Fig. 3 for a graphical illustration and Methods for a full derivation).

**Figure 4:**
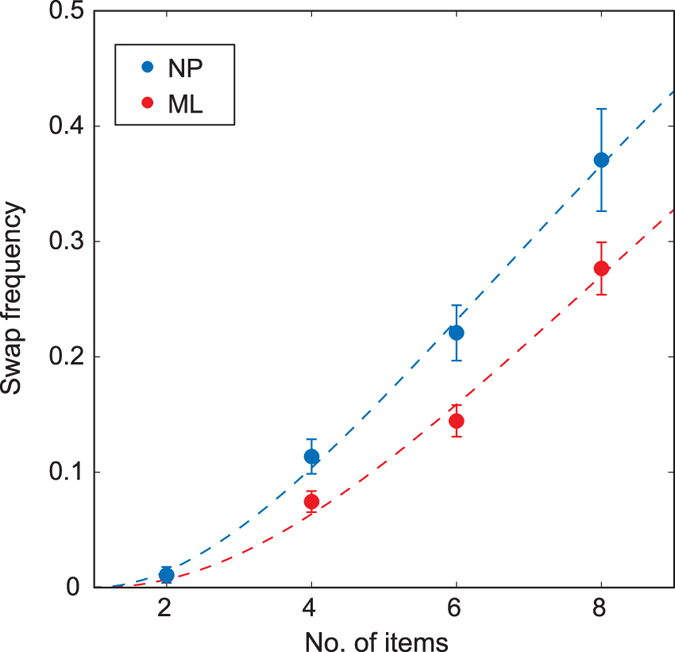
Swap error frequency estimated from empirical data (see Table 1) using the NP method (blue). Errorbars indicate ±1 SE. Probability of making a swap error increases with number of items in the memory array (set size). Estimates based on the NP method were consistently higher than those obtained by maximum-likelihood (red). Dashed lines indicate cumulative gamma functions that best fit the relationship between swap frequency and number of non-targets (no. of items −1).

The NP method was applied to a dataset combining results from 9 published studies that used a delayed report task to test memory for orientation, direction or colour (196 subject-feature pairs, >100,000 trials in total; see Table 1). NP estimates of swap frequency in this dataset are plotted in Fig. 4 (blue; ML estimates shown in red for comparison). The frequency of swap errors was found to increase monotonically with set size, from <2% of responses for 2 item arrays, up to 37% for 8 items (all p < 0.001). NP and ML estimates were correlated (r = 0.50, p < 0.001), but importantly the NP method identified a significantly greater frequency of swap errors for set-sizes 4 and above (all p < 0.001). Note that both sets of estimates agreed that the relationship between swap frequency and the number of non-targets was not linear, contrary to simplifying assumptions made by some previous ML models^12^,^22^. A cumulative gamma function (dashed blue curve) provided a better fit (p < 0.01), and has suitable values at extremes 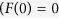, 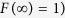.

**Figure 5:**
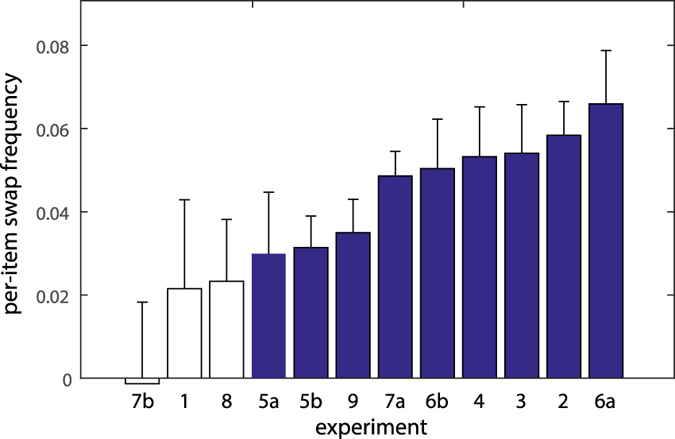
Per-item swap frequency based on largest set size tested (6 or 8 items) in each experimental dataset. Filled bars indicate values significantly (p < 0.05) greater than zero. Errorbars indicate ±1 SE.

Figure 5 shows how estimates of swap frequency varied between the 12 separate experiments making up the empirical dataset. Overall, 9 out of 12 experiments showed significant (p < 0.05) evidence for swap errors at the largest set size tested (either 6 or 8 items). There was one clear outlier, Exp. 7b, for which the estimate of swap frequency was approximately zero. This experiment was notable for having by some way the largest spatial separation between stimuli in the memory array, relative to their size, of any of the experiments examined (0.5° s.d. gabor stimuli, with a mean separation of 12.2°^11^). Over the set of experiments, there was a strong trend towards negative correlation between swap frequency and mean stimulus separation (r = −0.52, p = 0.085). This relationship between swap probability and spatial separation is explored more directly below.

**Figure 6:**
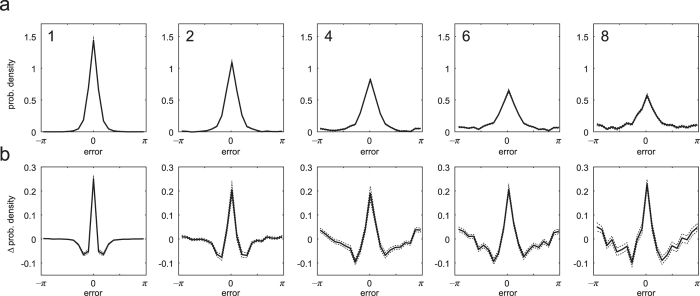
Estimates of *f* (*θ*), the distribution of errors in the report dimension. (**a**) Mean estimates of 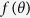 for different set sizes. Dashed lines indicate ±1 SE. (**b**) Deviation of 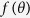 from a circular normal (Von Mises) distribution with the same variance. Distributions deviate significantly from normality at all set sizes.

Figure 6a plots NP estimates of the distribution of errors in the reported dimension, i.e. with the contribution of swap errors removed, for each set size. These plots are obtained by a simple method based on the histograms of responses calculated relative to each item in a display. The histogram of responses relative to the *k*th item arises probabilistically from a mixture of 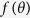 and the uniform distribution, in proportions 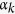 and 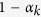 respectively. Because the mixture components 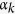 sum to one, summing up histogram values for the *m* items results in a mixture of 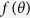 and the uniform distribution in the proportions 1 and 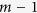, respectively. The uniform component, being a known proportion of the total, can then simply be subtracted to recover an estimate of 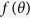.

**Figure 7:**
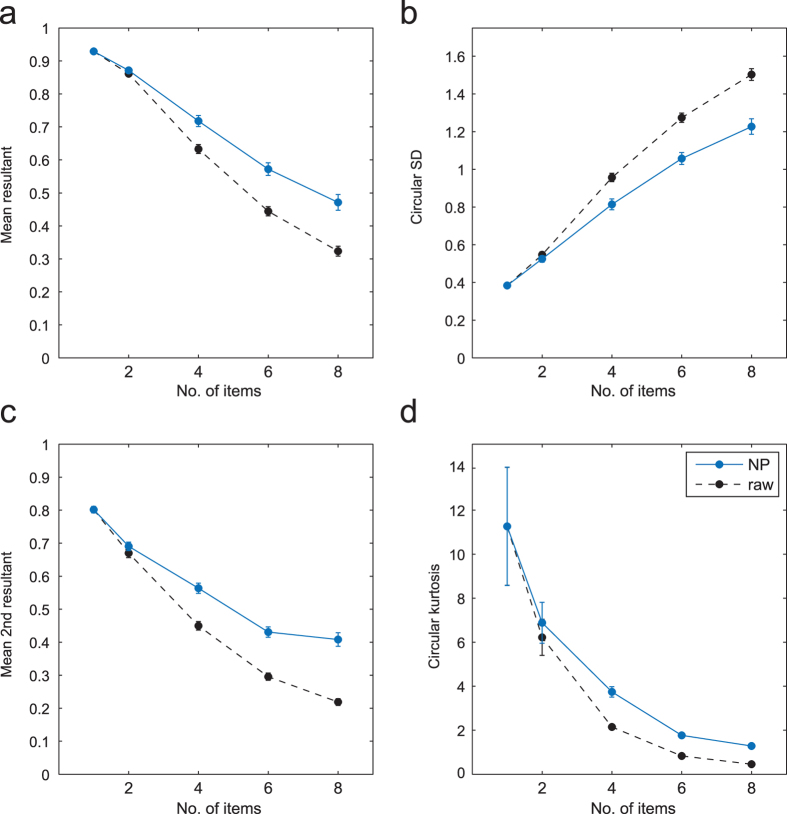
Estimated parameters of *f* (*θ*), the distribution of errors in the reported feature dimension. (**a**,**b**) Non-parametric estimates of mean resultant length (**a**) and circular standard deviation (**b**) of 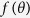 are plotted in blue. Variability in memory for the reported feature increases continuously as a function of set size. Raw parameters of the response error distribution (i.e. without any attempt to compensate for swap errors) are plotted for comparison (black). (**c**,**d**) Non-parametric estimates of mean second resultant (**c**) and circular kurtosis (**d**) of 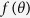 are plotted in blue; raw parameters in black. The distribution of errors in the reported feature dimension deviates significantly from normality at every set size (the circular normal has kurtosis around zero).

Figure 7a (blue) plots the NP-estimated *mean resultant length* of the error distribution (a measure of dispersion) and Fig. 7b the corresponding circular standard deviation. Shown in black are the same parameters calculated directly from the distribution of responses around the target feature. The lower error variability obtained using the NP method (n = 2, p = 0.078; n > 2, all p < 0.001) confirms that swap errors (which are randomly-distributed relative to the target) contributed significantly to the overall dispersion of responses about the target feature. Removing the influence of swap errors revealed the underlying distribution of error in the reported feature dimension: this distribution displayed a steady increase in variability with each increase in the size of the memory array (Fig. 7b; all p < 0.001).

As can be seen in Fig. 6a, errors were not distributed according to a normal distribution, even once swap errors had been removed: qualitatively, the distributions had sharper peaks and longer tails. Figure 6b plots the mean discrepancy between NP-estimated distributions and circular normal (Von Mises) distributions with the same mean and variance. These plots show that deviations from normality were observed at all set sizes, and were not due to averaging across participants with different error variabilities. One measure used to assess normality is the circular kurtosis, plotted in Fig. 7d. NP-estimated kurtosis (blue) deviated significantly from zero at every set size (all p < 0.001), indicating non-normality.

Surprisingly, kurtosis once swap errors had been removed (blue) was higher than the raw kurtosis of responses about the target (black). Naïvely, we might have predicted that the presence of randomly-distributed swap errors would increase non-normality of the error distribution, whereas these results seem to indicate the opposite. In fact, the addition of a uniform (random) component can *decrease* the kurtosis of a distribution, if the distribution has positive kurtosis to begin with. Note that kurtosis, based on the second circular moment of the distribution (Fig. 7c), is just one measure of the normality of a distribution: different effects might be observed on other measures or higher moments.

**Figure 8:**
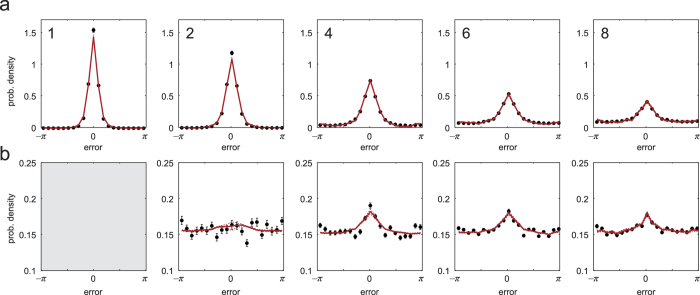
Validation of non-parametric estimates. Black symbols indicate empirical distribution of response deviations from the target feature value (**a**) and non-target feature values (**b**). These raw responses confound variability in the report dimension with swap error frequency. Red curves indicate response probabilities calculated from non-parametric estimates of swap frequency (Fig. 4) and 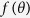 (Fig. 6).

To validate the results of the NP analysis, I examined the distribution of response deviations from target (Fig. 8a) and non-target (Fig. 8b) feature values (experimental data plotted in black). Red curves show the corresponding distributions generated from the NP estimates of swap frequency (Fig. 4) and report-dimension error distribution (Fig. 6). If the NP estimates are accurate, and the generative model illustrated in Fig. 1b is appropriate, these computed distributions should reproduce the empirical data (note, this test is similar to examining residuals of a fitted model in order to assess the success of the fitting procedure and the appropriateness of the model).

The computed distributions closely reproduced the pattern of empirical deviations from the target at each set size (Fig. 8a) (normalized MSE: for 2 items, 0.001; 4 items, 0.010; 6 items, 0.012; 8 items, 0.041). Figure 8b plots response deviations from non-target features at each set size. Models that do not incorporate the possibility of swap errors (e.g.^6^,^11^) predict that these distributions will be uniform. Instead, a significant central tendency was observed at each set size (V test: 2 items, p = 0.028; 4 items, p < 0.001; 6 items, p < 0.001; 8 items, p < 0.001), confirming the presence of swap errors. The distributions computed from NP estimates (red) reproduced these central tendencies. Here, accuracy notably increased with set size (normalized MSE: for 2 items, 1.3; for 4 items, 0.35; for 6 items, 0.13; for 8 items, 0.16). Finally, applying the ML method to simulated data drawn from the NP-computed distributions showed the expected underestimation of swap error frequency (see Supplementary Table S1).

**Figure 9:**
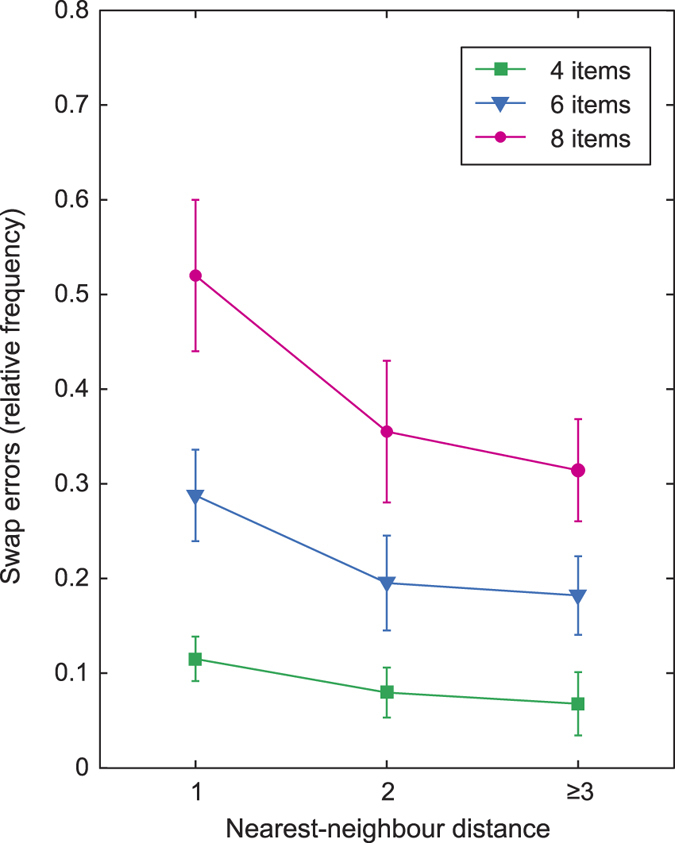
Probability of incorrectly reporting a non-target item as a function of its proximity (nearest-neighbour distance) to the target item. Nearest neighbours are significantly more likely to be erroneously reported than items further from the target. Probabilities are normalized to account for unequal frequency of items at different distances.

NP estimates of swap frequency can be obtained for arbitrary subsets of array items presented over a sequence of trials, i.e. we can estimate the probability that one of a particular group of items gets reported in place of the target. One factor that could influence swap probability is an item’s similarity to the target in the *probe* feature space, i.e. spatial proximity, in the case of location probes. Figure 9 plots NP-estimated swap frequency as a function of a non-target’s nearest-neighbour (NN) distance to the target (note that, due to symmetries in the arrangement of stimuli, nearest-neighbour distances greater than 3 were rare, and so are grouped with distance 3 for this analysis; Studies 1 and 9, for which spatial information was not available, and Study 8, which probed by colour, were excluded).

The results indicate that the non-targets in closest spatial proximity to the target were significantly more likely to be the subject of swap errors than more distant non-targets (NN distance 1 vs 2: p = 0.019; 1 vs ≥3: p = 0.003; 2 vs ≥3: p = 0.58). In agreement with results above, swap errors increased significantly with set size at each NN distance (all p ≤ 0.001). Interactions between distance and set size were not significant (p > 0.5; note that metric distances between items at the same nearest-neighbour distance declined on average with set size.).

## Discussion

This study assessed the frequency of swap errors in visual memory by applying novel non-parametric methods to data from delayed estimation tasks. Previous swap estimates have been inextricably tied to particular assumptions about how errors are distributed in the reported feature dimension. As this distribution is *a priori* unknown, and the canonical (normal) distribution appears not to provide a good description of it, previous estimates of swap frequency have necessarily been provisional. In contrast, the NP methods described here make no assumptions about the shape of this distribution. The results suggest that swap errors may occur with higher frequency than previously appreciated, accounting for more than a third of trials (37%) at set size 8.

Maximum likelihood (ML) estimators have a number of valuable properties, in particular their statistical efficiency. In the present situation, simulations indicated that *with the correct model* the ML method could achieve the same variability as the NP method using roughly one quarter the data (see Supplementary Figure S2). However, there is little benefit to being precisely wrong, and I found that ML estimates of swap frequency were strongly biased if the model of within-dimension variability 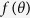 did not match the ground truth. Contrastingly the NP estimates, while more variable for a given amount of data, did not depend for their reliability on a correct specification of 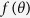.

An additional advantage over ML is that the NP method does not require computationally-costly non-linear optimization techniques, which sometimes fail to converge, or converge to the wrong value (i.e. a local maximum). This consideration tends to limit the number of free parameters incorporated into a model: some previous studies^12^,^22^ may have underestimated swap frequency at higher set sizes because they assumed an overly-simplistic linear relationship between number of non-targets and swap probability. The NP method could prove useful beyond the domain of working memory; potentially in any situation where the mixture model described in Fig. 1b is appropriate. To give one example, a type of swap error (termed an “illusory conjunction”^23^) can cause visual stimuli to be misperceived under certain circumstances: when presentation is very brief, attention is directed elsewhere, or under conditions of crowding^24^. With a suitable experimental design, the NP method could prove a useful tool for investigating these errors of perception.

Although it makes far fewer assumptions than previous methods, it should be emphasized that NP is not model-free. The main assumption, shared with most previous approaches, is that errors in the report feature dimension are independent of errors in selecting which item to report (see Fig. 1b). This could be violated in several ways. If, as the present results suggest, swap errors are the result of variability in the probe feature dimension, then a correlation between report- and probe-dimension error magnitudes would violate this assumption of the model. However, previous studies that have examined recall of multi-feature objects found no correlation between errors in different dimensions^15^,^25^,^26^.

Another way in which the model underlying NP could be incomplete is if responses were biased towards the average feature value of presented items, as has been observed in some studies^27^,^28^; however, a previous meta-analysis^22^ found no evidence for this kind of bias in experiments where all stimuli were, as here, selected uniformly at random from the same parameter space. Yet another potential source of error would be if observers swapped in items that were similar to the target in the report dimension: there is currently no evidence for such errors, but neither NP nor current ML methods would be expected to detect them. The NP method should be robust against stimulus-specific variations in memorability as reported by some studies^29^,^30^. Further evidence that the simple model in Fig. 1b captures at least a large proportion of behaviour on this kind of task is that responses generated under the model can quite accurately reproduce the observed distributions of error deviations from both target and non-target features (Fig. 8). However, there are some deviations from these predictions at lower set sizes that might indicate additional factors at work.

A subtle but important distinction between NP and ML methods concerns how they treat random responses. The ML method of Bays, Catalao, and Husain^8^ attempts to assign response frequencies to three bins: target, swap, and random (uniform). An implicit assumption of this method is that a swap error cannot result in a random response. In contrast, the NP method treats any random responses present in the data as part of 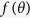, with the result that they are expected to swap with the same frequency as other responses. If this assumption is wrong, the NP method may overestimate swap frequency. The present results seem to support this assertion of the NP model, in that they indicate that swap errors arise from uncertainty in recall of probe-dimension features (see below for a detailed discussion). In which case, if the report-dimension features of items are sometimes forgotten, we should expect random responses to be swapped in for the correct response at the same rate as other non-target responses (noting that errors in different feature dimensions occur independently^15^,^25^). The tendency to assign responses to a uniform component which cannot swap may contribute to the ML method’s lower estimation of swap frequency when the model does not fit the ground truth. This would remain a consideration even if the ML method were equipped with the correct distribution of errors in the report dimension. It remains an empirical question as to whether there exist truly random responses in analogue report data (as opposed to very imprecise responses) and how such responses are generated.

The original mechanism put forward for swap errors^8^ was that variability in recall of the probe-dimension features in an array (in that case, item locations) would result in occasional incorrect matches between the probe and the items in memory, and hence reports of the wrong item. This account made the strong prediction that these errors would be most likely for non-targets with probe-features most similar to the target’s. However, at the time it was not possible to test that prediction, as attempts to fit an ML model with the necessary additional parameters failed. The present results, based on a non-parametric method, confirm that a target’s nearest neighbours in space are significantly more likely to be mistaken for the target of a spatial probe.

A study that required observers to report two different visual features (colour and orientation) of one item indicated by a spatial probe^15^, found that swap errors occurred *independently* in each feature dimension. At first this seems contrary to a spatial-variability account of swap errors, in that one would expect an observer, having mis-identified a non-target item as matching the probe, to report both features of that same item. One possible resolution to this paradox is that the two features are retrieved sequentially, with each retrieval requiring a separate comparison between the noisy memories of location and the probe^31^.

Alternatively, the probe might be simultaneously compared to two independent representations of item locations in the brain: one storing the conjunction of colour and location information, the other the conjunction of orientation and location. This latter proposal has neurophysiological plausibility, as neurons selective for simple visual features are almost without exception found to have spatial selectivity as well (i.e. a spatial receptive field).

The present results are consistent with conclusions of two previous studies using alternative methods. Emrich and Ferber^32^ used the ML method to estimate swap frequency in a task in which the spatial separation of items was manipulated. They observed an overall increase in estimates of swap frequency when array items were closer together. However, their method could not identify which non-targets were the source of incorrect reports, and hence could not rule out a more general effect of visual crowding^24^. In contrast the present study explicity demonstrates that non-targets most proximal to the probe are most likely to be misidentified as the target. Rerko, Oberauer, and Lin^31^ also found evidence for effects of spatial similarity on error frequency in a recall task, in which stimuli and responses were selected from a small group of dissimilar colours. As with the related *change detection* method^33^, it is challenging in this kind of task to distinguish swap errors from errors due to variability in the report dimension (colour); however the increase in misreport frequency with distance is consistent with the present findings.

Results of the current study have important implications for the study of binding representations. Previously, binding in visual working memory has predominantly been studied using a modification of the standard change detection task, in which subjects compare two sequential arrays of items (e.g. coloured shapes) and judge whether they are the same or different. Memory for binding is tested in a special condition in which the two arrays contain an identical set of features, but differ in the conjunction of those features (e.g. a red circle and a blue triangle is replaced by a blue circle and a red triangle). Performance in this conjunction condition is typically above chance, but impaired compared to detecting a simple feature change, and this has generally been interpreted as indicating a fallible or limited-capacity storage mechanism specifically for binding information^14^. Greater impairment in the conjunction condition as a result of Alzheimer’s^19^ or cognitive aging (34; but see^18^,^35^) has implicated failure of the binding mechanism in these conditions.

The current results present a different perspective on binding errors, demonstrating that noise in memory for spatial location can result in confusion between visual features. Analogously, uncertainty in the representation of locations corresponding to the different features in the sample array could be responsible for impairment in the conjunction condition of the change detection task, i.e. noise in location memory could cause features from different objects to be incorrectly judged as coinciding in space and hence belonging to the same object. This would make the conjunction condition an indirect assessment of spatial memory precision, rather than binding. The impairments observed in patient groups could likewise reflect deficits in spatial recall; this possibility could be tested using sensitive measures of location memory precision (e.g.^5^).

In addition to estimates of swap frequency, NP methods were used to recover the distribution of errors within the reported feature dimension. This is necessary because the raw deviation of responses from targets confounds this underlying distribution with swap errors, which appear uniformly distributed relative to the target. ML methods cannot provide an unprejudiced estimate of the true distribution, because they require it to be specified in advance up to a small number of free parameters. The nature of this distribution and its changes with set size are considered vital pieces of evidence for understanding the limits on working memory. In many cases, the distinction between competing models hinges on small differences in parameters including standard deviation and kurtosis, which are shown here to be distorted by swap errors. While discriminating between the various competing models is not the main purpose of this study, some observations resulting from the present analysis may prove valuable.

First, this analysis confirms one of the most important new findings from analogue report tasks: that the variability with which a single visual feature can be recovered from working memory increases steadily and continuously with the total set size, or memory load (Fig. 7b). Swap errors, because they are randomly distributed relative to the target, contribute significantly to the raw deviation of responses as set size increases, but their exclusion does not alter this fundamental relationship. There is no evidence in this measure for any discontinuity that might reflect a deterministic limit on the number of items in memory.

Second, while swap errors distort the distribution of raw errors, excluding them did not reveal the underlying distribution to be normal (Fig. 6). The observation of long-tails in the raw errors at higher set sizes has been interpreted as evidence of guessing, due to exceeding a fixed capacity limit^6^. Bays, Catalao, and Husain^8^ showed that, once swap errors were accounted for (by ML methods), the empirical distributions were no longer consistent with this model. This is also confirmed by the present results, which show that the underlying error distribution deviates from normality at every set size, including 1 item. Conceivably, separate mechanisms could be responsible for non-normality above and below the putative capacity limit, but any such account would have to explain why these mechanisms have so arranged themselves that there is no perceptible discontinuity at capacity in standard measures of distribution shape, such as kurtosis (Fig. 7d).

A parsimonious model would invoke a single mechanism to explain non-normality at every set size. One such proposal^12^ is based on the principles of neural coding believed to underlie sensory representations in cortex^36^. This study found that feature values retrieved from such *population codes* display deviations from normality matching those observed in experimental data. While incorporating a probability of swap errors into this model improved its fit to data, the population code was restricted to representing the reported feature dimension. However, a closely-related model^37^, also based on population coding, has recently been shown to qualitatively reproduce swap errors on recall tasks, by incorporating neurons that represent the conjunction between report and probe features. Currently no model simultaneously predicts the deviations from normality demonstrated in Fig. 6 and the swap error frequencies revealed in Fig. 4, so this remains an important goal for future research.

## Methods

### Experimental procedure & generative model

Consider behavioural experiments of the kind exemplified in Fig. 1a. On the *j*th of *n* trials a visual array is presented consisting of *m* items with feature values (in this example, orientations) 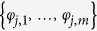. The feature values are independently chosen at random from a uniform distribution on a circular space 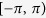, i.e.


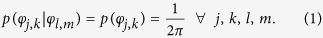


After a brief delay, the observer is instructed to report one of the items in memory, as indicated by a probe in a second feature dimension (in this example, location), and generates response 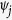. To account both for dispersion in recall of the test features about their true values, and the possibility of incorrectly reporting one of the unprobed items, we model responses with a mixture comprising an unknown distribution 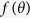 and mixture parameters 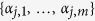:


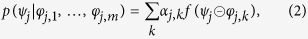


where 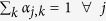 and 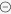 indicates subtraction on the circle. See Fig. 1b for a graphical illustration. We would like to estimate mixture parameters and the unknown distribution 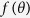 from empirical response distributions, and more specifically we will make use of the response errors calculated relative to each item value, i.e.


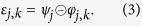


### Estimating the mean mixture parameter for a subset of items, 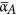


Consider a subset *A* of all memory items presented 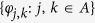 (for example, these could be all the probed items in a sequence of trials). We wish to estimate the mean mixture parameter for items in *A*,


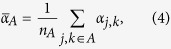


where 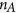 is the number of items in *A*. Defining:


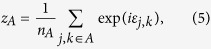



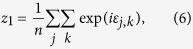


our estimator of 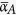 is


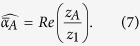


In geometric terms, 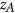 is the mean resultant vector of errors calculated relative to items in *A*, 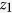 is the vector sum of the mean resultant vectors of errors calculated relative to all items, and 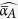 is the ratio of the magnitude of the component of 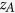 in the direction of 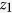, to the magnitude of 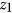. Taking the ratio of the component in the direction of 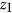 (equivalent to the real part of the ratio) was found to provide a more reliable estimator than a simple ratio of moduli; under the generative model, 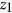 and 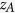 have the same direction, so any component orthogonal to 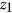 (imaginary part) is due to noise.

### Proof of consistency

From (1), (2) and (3), the probability that error 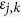 takes value *θ* is given by,


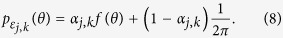


So,


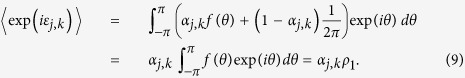


where 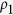 is the first circular moment (resultant vector) of 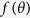 and 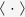 denotes expectation. By the law of large numbers,


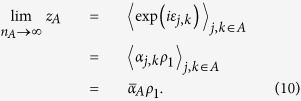


Similarly,


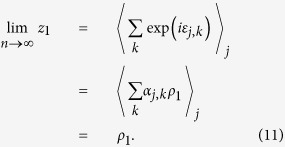


So, considering that 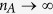 implies 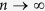,


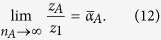


### A note on bounds

The non-parametric estimate occasionally falls outside the bounds of interpretable values, i.e. the range of probabilities [0, 1]. This is a desirable property: a bounded estimator, unless it is perfectly accurate, is necessarily biased in proximity to its bounds. However, a large out-of-bounds estimate can excessively influence group means. A compromise is to constrain the estimator beyond the true range: bounding at [−1, 2] was found in simulations to provide a good balance between bias and variance for this estimator (see Supplementary Figure S2).

### Estimating the true error distribution in the report dimension, *f* (*θ*)

Define 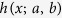 as an indicator function taking value 1 if *x* falls between *a* and *b* on the circle, and 0 otherwise. We can estimate 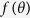 by


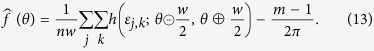


### Proof of consistency


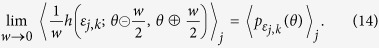


So,


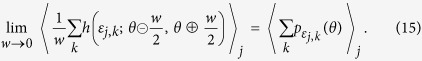


From (8),


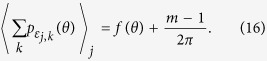


So, by the law of large numbers,


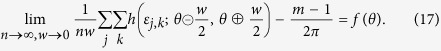


### Estimating circular moments of error in the reported feature dimension

The 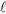
th circular moment of 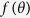 can be estimated by


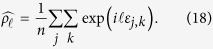


### Proof of consistency

From (8),


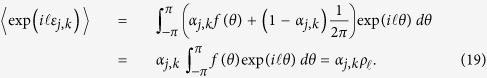


So, by the law of large numbers,


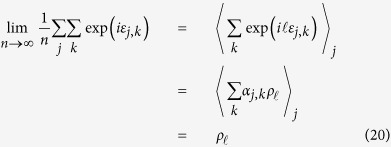


### Simulations

I examined the ability of the non-parametric (NP) estimator (Eq. 7) to identify the frequency of swap errors (i.e. reporting an item other than the one that was probed) in simulated datasets. Performance was compared to a previous method for estimating swap frequency^8^ based on maximum-likelihood (ML) fitting of a particular parametric model to data. Specifically, the model of errors in the report dimension, originating with Zhang and Luck^6^, comprises a probabilistic mixture of a circular normal (Von Mises) and a uniform distribution (see Bays, Catalao, and Husain^8^, for full details). To provide a fair comparison with the NP method, which does not explicitly incorporate a uniform component, ML swap estimates are reported as a fraction of non-uniform responses.

Each simulation consisted of 50, 100, 200, 400 or 800 artificial trials, with 2, 4 or 8 items presented per trial. Responses were simulated according to the generative model illustrated in Fig. 1b. The probability of reporting the target item was fixed at 0.95, 0.8 or 0.5, with the remaining reports distributed equally between non-target items. With respect to the distribution of errors in the report dimension, 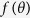, the simulations fell into two classes: *normal* and *arbitrary*. In the *normal* case, 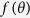 corresponded to a Von Mises distribution with mean zero and concentration parameter 2, 10, or 50. Data from these simulations was therefore consistent with the assumptions of the ML model. In the *arbitrary* case, 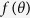 was generated by a random mixture of between 1 and 4 Von Mises functions, each with randomized means and concentrations, with the requirement that the resulting distribution have circular s.d. ≤1, matching the *normal* simulations. In general, data from these simulations was *not* consistent with the assumptions of the ML model. The extent of this deviation was measured by the Hellinger distance between each *arbitrary* distribution and a variance-matched zero-mean Von Mises. A different 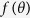 was generated for each dataset; 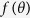 was consistent for all trials within a dataset. Simulated datasets numbered in total 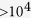.

### Experimental data

I examined data from nine published studies of visual working memory (see Table 1 for summary; data from studies 1–3 and 6–8 were previously collated and made publicly-available by van den Berg, Awh, and Ma^22^; one study from this previous set was excluded because non-target values could not be unambiguously recovered). Where more than one target feature was tested in the same experiment, each subject × feature combination was treated as an independent dataset. Where there were other experimental variations, e.g. in method of response, these were disregarded and the data pooled. The studies differed in which array sizes were tested: to avoid relying too heavily on results from any single study, I restricted my analysis to the set sizes 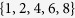, which were most consistently represented across the different studies. In total, the dataset comprised 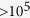 trials. ANOVA and *t*-tests were used for hypothesis testing.

The studies varied considerably in the number of trials each subject completed at each set size (rightmost column in Table 1), and hence in the reliability of the estimates obtained for each subject. Simulations indicated that variance of both NP and ML estimators had an approximately 1/*n* relationship with number of samples, so to take these differences into account optimally when estimating global means and other descriptive statistics, I weighted each observation in proportion to the number of trials on which it was based.

## Additional Information

**How to cite this article**: Bays, P. M. Evaluating and excluding swap errors in analogue tests of working memory. *Sci. Rep.* **6**, 19203; doi: 10.1038/srep19203 (2016).

## Supplementary Material

Supplementary Information

## Figures and Tables

**Table 1:** Experimental studies.

No	Study	Report	Probe	Subjects	Trials
1	Zhang & Luck, 2008	Color	Location	8	125
2	Bays *et al.*, 2009	Color	Location	12	200
3	Anderson *et al.*, 2011 (Exp 1)	Direction (360 °)	Location	45	120
4	Bays, Gorgoraptis *et al.*, 2011	Orientation	Color	32	800
5a	Bays, Wu & Husain, 2011	Orientation	Location	10	300
5b	Bays, Wu & Husain, 2011	Color	Location	10	300
6a	Anderson & Awh, 2012 (Exp 3)	Orientation	Location	23	120
6b	Anderson & Awh, 2012 (Exp 3)	Direction (360 °)	Location	23	120
7a	Van den Berg *et al.*, 2012	Color	Location	13	216
7b	Van den Berg *et al.*, 2012	Orientation	Location	6	320
8	Rademaker *et al.*, 2012	Orientation	Location	6	800
9	Bays, 2014 (Exp 1)	Orientation	Location	8	225
