# Supplementary material for: Evaluating and excluding swap errors in analogue tests of working memory
Source: Sci Rep. 2016 Jan 13;6:19203. doi: 10.1038/srep19203 (PMC4725843; doi:10.1038/srep19203)
Supplement: Supplementary Information [file srep19203-s1.pdf]

Evaluating and excluding swap errors in analogue report

Paul M Bays

Supplementary material

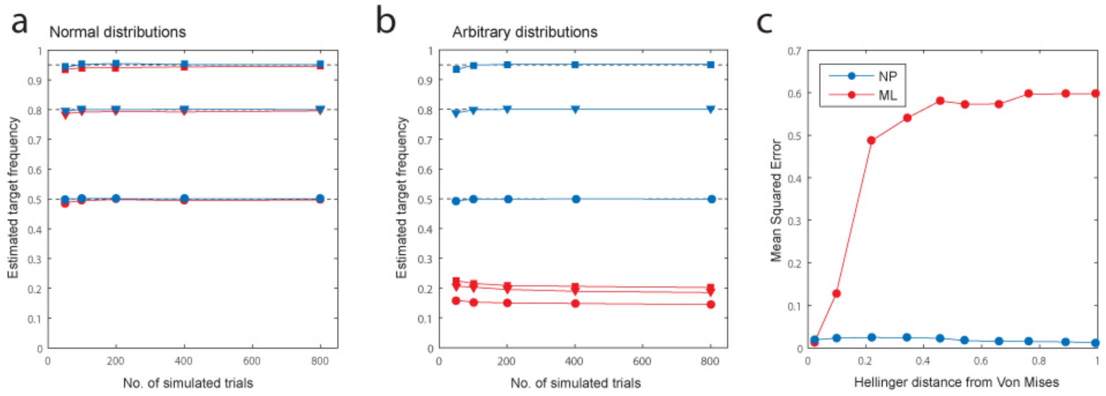

Supplementary Figure S1: Comparison of ML and NP estimates of target response frequency, based on simulated recall data. (a) Simulations in which errors in the report dimension $f(\theta)$ are drawn from a circular normal (Von Mises) distribution. True frequencies: 0.5, circles; 0.8, triangles; 0.95, squares). (b) Simulations with arbitrary (randomly-generated) distributions $f(\theta)$. (c) Estimation error as a function of similarity of $f(\theta)$ to Von Mises.

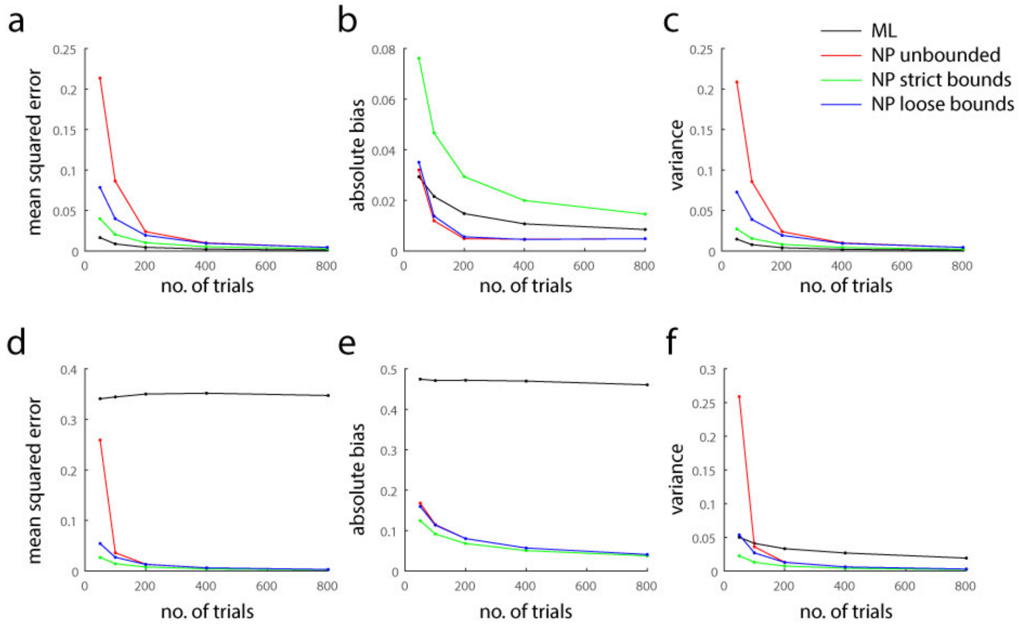

Supplementary Figure S2: Analyses of bias and variance. Performance of different methods in estimating swap frequency from simulations based on (a–c) the ML generative model and (d–f) arbitrary distributions (see Methods). Note that the unbounded NP method produces highly variable estimates for smaller numbers of trials (red curves in c & f). Placing strict bounds (at [0, 1]) on NP estimates reduces variance but results in strongly biased estimates in some circumstances (green curve in b). Loose bounds at [–1, 2] were found to provide a consistent stable trade-off between bias and variability (blue curves).

No of items	2	4	6	8
True p(swap)	0.0110	0.1135	0.2208	0.3707
NP estimate	0.0185	0.1034	0.2332	0.3637
ML estimate	0.0142	0.0888	0.1378	0.1805

Supplementary Table S1: Mean estimates of swap probability resulting from NP and ML methods applied to 1000 simulated datasets of 200 trials drawn from the mean estimated NP generative model shown in Fig 8 (red curves). Note that the ML method strongly underestimates swap frequency, consistent with observations in the main text.
